# Absence of *sodA* Increases the Levels of Oxidation of Key Metabolic Determinants of *Borrelia burgdorferi*

**DOI:** 10.1371/journal.pone.0136707

**Published:** 2015-08-31

**Authors:** Maria D. Esteve-Gassent, Trever C. Smith, Christina M. Small, Derek P. Thomas, J. Seshu

**Affiliations:** 1 South Texas Center for Emerging Infectious Diseases, The University of Texas at San Antonio, San Antonio, TX-78249, United States of America; 2 Center of Excellence in Infection Genomics, The University of Texas at San Antonio, San Antonio, TX-78249, United States of America; 3 Department of Biology, The University of Texas at San Antonio, San Antonio, TX-78249, United States of America; 4 Department of Veterinary Pathobiology, College of Veterinary Medicine, Texas A&M University, College Station, TX-77843, United States of America; University of Kentucky College of Medicine, UNITED STATES

## Abstract

*Borrelia burgdorferi*, the causative agent of Lyme disease, alters its gene expression in response to environmental signals unique to its tick vector or vertebrate hosts. *B*. *burgdorferi* carries one superoxide dismutase gene (*sodA*) capable of controlling intracellular superoxide levels. Previously, *sodA* was shown to be essential for infection of *B*. *burgdorferi* in the C3H/HeN model of Lyme disease. We employed two-dimensional electrophoresis (2-DE) and immunoblot analysis with antibodies specific to carbonylated proteins to identify targets that were differentially oxidized in the soluble fractions of the *sodA* mutant compared to its isogenic parental control strain following treatment with an endogenous superoxide generator, methyl viologen (MV, paraquat). HPLC-ESI-MS/MS analysis of oxidized proteins revealed that several proteins of the glycolytic pathway (BB0057, BB0020, BB0348) exhibited increased carbonylation in the *sodA* mutant treated with MV. Levels of ATP and NAD/NADH were reduced in the *sodA* mutant compared with the parental strain following treatment with MV and could be attributed to increased levels of oxidation of proteins of the glycolytic pathway. In addition, a chaperone, HtpG (BB0560), and outer surface protein A (OspA, BBA15) were also observed to be oxidized in the *sodA* mutant. Immunoblot analysis revealed reduced levels of Outer surface protein C (OspC), Decorin binding protein A (DbpA), fibronectin binding protein (BBK32), RpoS and BosR in the *sodA* mutant compared to the control strains. Viable *sodA* mutant spirochetes could not be recovered from both gp91/*phox* ^*−⁄−*^ and iNOS deficient mice while borrelial DNA was detected in multiple tissues samples from infected mice at significantly lower levels compared to the parental strain. Taken together, these observations indicate that the increased oxidation of select borrelial determinants and reduced levels of critical pathogenesis-associated lipoproteins contribute to the *in vivo* deficit of the *sodA* mutant in the mouse model of Lyme disease. This study, utilizing the *sodA* mutant, has provided insights into adaptive capabilities critical for survival of *B*. *burgdorferi* in its hosts.

## Introduction

*Borrelia burgdorferi*, the causative agent of Lyme disease is transmitted to humans by the bite of infected *Ixodes* spp. ticks [1]. Due to the highly disparate nature of the environmental signals present in the tick vector before and after a blood meal, *B*. *burgdorferi* exhibits rapid adaptive gene expression in response to these cues [2–5]. Some of these signals include differences in temperature, pH, levels of dissolved gases, reactive oxygen and nitrogen species (ROS/RNS) and a variety of other nutrients resulting in significant changes in growth characteristics of the spirochetes in the tick mid-gut following the ingestion of a blood meal [6–8]. The alterations in the physiology/metabolism of *B*. *burgdorferi*, in turn, enable the spirochetes to migrate from the tick mid-gut to the salivary glands facilitating transmission, colonization and dissemination to and within vertebrate hosts [9–12]. While a large body of information exists on the variety of lipoproteins critical for the initial stages of infection, the role of non-specific effects of factors present in the incoming blood meal and tick saliva affecting the kinetics of transmission of *B*. *burgdorferi* are beginning to be understood in greater detail [4, 6, 13–18].

We previously reported that the inactivation of the gene encoding superoxide dismutase A (*sodA*) in *B*. *burgdorferi* resulted in a mutant strain that could not be re-isolated from infected tissues following intradermal needle inoculation in C3H/HeN mice at 21 days post-infection [19]. Additional studies employing these strains have expanded the significance of *sodA* within the context of borrelial physiology ([20] The *sodA* deficient strain was also more susceptible to the killing effects of activated macrophages and neutrophils compared to the wild type or complemented strains [19, 21]. Components of the tick saliva with anti-oxidant properties have also been shown to enhance the survival capabilities of *B*. *burgdorferi* following transmission from the ticks suggesting that multiple borrelial and vector specific components contribute to the resistance against the mediators of innate immune response in the mammalian host [17, 22–24]. While the *sodA* mutant did not have a significant growth defect under *in vitro* growth conditions, we analyzed the contributions of the levels of oxidation and synthesis of select borrelial proteins that contributed to reduced survival capabilities of the *sodA* mutant under *in vivo* conditions.

The deleterious effects of reactive oxygen species (ROS) on the survival of several bacterial pathogens have been attributed to DNA damage induced via the interaction of hydrogen peroxide with “free” Fe^2+^ resulting in highly reactive OH (Fenton reaction) [25]. Previous studies have shown that the intracellular levels of free Fe in *B*. *burgdorferi* are not sufficient to sustain a robust Fenton reaction and that DNA is not the primary target of ROS [26, 27]. Borrelial membranes, which incorporate polyunsaturated lipids from either the host or from *in vitro* growth medium, were identified as the primary target for ROS [26, 27]. These aforementioned studies identified linoleic acid as a major target for ROS in the wild type *B*. *burgdorferi* strain B31-A3. In addition, an increase in the level of end products from the oxidation of polyunsaturated fatty acids and detectable changes to the borrelial membrane architecture was observed [26]. Moreover, the accumulation of damage to a variety of biomolecules and structures could eventually contribute to increased sensitivity of *B*. *burgdorferi* to reactive oxygen and reactive nitrogen species (ROS/RNS) [28]. Nitric oxide toxicity in *B*. *burgdorferi* leads to alteration in free and zinc-bound cysteine thiols notably zinc-dependent glycolytic enzyme fructose-1, 6-bsiphosphate, Borrelia oxidative stress regulator (BosR) and neutrophil activating protein (NapA). BosR and NapA were specifically shown to be S-nitrosylated in borrelial cells exposed to NO donor diethylamnine NONOate (DEA/NO) [28]. Collectively, alterations affecting the structure/function of key borrelial proteins could have a significant impact on the survival of the spirochetes in different microenvironments where the levels of these stressors are elevated, such as in the vicinity of activated macrophages and neutrophils in the vertebrate hosts.

A number of studies have determined the contributions of bacterial superoxide dismutases (Sods) in the colonization capabilities of the respective bacterial pathogens in different experimental animal models (reviewed in [29]). Several bacterial pathogens possess multiple Sods and the absence of Mn-SodA may not confer a significant reduction in the colonization capabilities in animal models. However, deletion of SodA in *Yersinia enterolitica* serotype O8 resulted in mutants with a significant reduction in tissue colonization [30]. Since *B*. *burgdorferi* has a single Sod and has a paucity of antioxidant defense mechanisms even though the intracellular levels of free iron is low, it is feasible that host-derived ROS and RNS can limit the survival of *sodA* mutant via the interaction of NO and superoxide. While the ROSs are produced by macrophage NADPH oxidase (Phox), RNS is generated via the inducible nitric oxide synthase (iNOS). Therefore, we rationalized that mice lacking the heme-binding subunit of the superoxide generating NADPH oxidase, gp91/phox^*−⁄−*^ [31] or iNOS [32, 33] could potentially allow for the colonization *sodA* mutant and provide a basis for the inability of *sodA* mutant spirochetes to survive in C3H/HeN mice.

In this study, we hypothesized that lack of SodA leads to increased levels of intracellular O_2_^–^ in *B*. *burgdorferi* and result in changes in key borrelial proteins ultimately reducing the survival of the *sodA* mutant in mouse models of infection. We undertook a proteomic approach to identify the proteins with increased susceptibility to oxidation in the absence of SodA [34–36]. A subset of proteins of the glycolytic pathway were found to be oxidized at a higher level in the *sodA* mutant compared to the parental wild type strain in the presence of 20 mM MV. A concomitant reduction in the levels of ATP/NADH was also observed in the *sodA* mutant reflecting reduced function of select enzymes in the glycolytic pathway. Mutant strains of mice either lacking gp91/phox^–/–^ or iNOS were unable to support the survival of the *sodA* mutant. Additional phenotypic analysis of the *sodA* mutant revealed that multiple lipoproteins critical for colonization of the vertebrate host by *B*. *burgdorferi* were reduced in the *sodA* mutant compared to the parental and complemented strains. T Regulators of gene expression such as RpoS and BosR were reduced in the *sodA* mutant that could partly contribute to the reduced levels of select lipoproteins thereby limiting survival of the *sodA* mutant in the vertebrate hosts. Proteomic analysis of borrelial mutants serves as a tool to dissect the molecular basis for the *in vitro* and *in vivo* phenotype, which can be readily applied to a growing list of borrelial mutants with a wide-range of colonization and infectivity capabilities.

## Materials and Methods

### Ethics statement

All animal experiments were done using protocols approved by the Institutional Animal Use and Care Committee (IACUC) at The University of Texas at San Antonio.

### Bacterial Strains and Growth conditions

A clonal isolate of *B*. *burgdorferi* strain B31 lacking lp25 transformed with pBBE22 (ML23/E22) and a mutant strain lacking *sodA* (*sodA* ^*–*^ */*E22) as previously described were cultured in BSK-II medium supplemented with 6% normal rabbit serum (Pel-Freez, AK) at 32°C in a CO_2_ incubator with 1% CO_2_ [19, 37–39]. The spirochetes were grown to a density of 5 x10^7^ spirochetes/ml and washed thrice in HBSS/5mM Ca_2_Cl/50mM sucrose/2%bovine serum albumin and was subjected to oxidative stress by treating with 20mM MV, an endogenous O_2_^–^ generator for a period of 1 hr in the aforementioned buffer [19]. Oxidative stress experiments were done in triplicate, and all replicates were processed for proteomic analysis as described below.

### Preparation of Protein Fractions

After the induction of oxidative stress for the indicated time, the spirochetes were washed to remove MV and bovine serum albumin with 25mM NaCl + 5mM DTT + 20mM HEPES pH 7.6 containing Complete protease inhibitor (Roche) and lysed by sonication. The cell lysates were cleared by centrifugation for 15 minutes at 10,000*g* and 4°C to remove intact bacteria and the membrane, and soluble proteins were separated by ultracentrifugation at 150,000x*g* for 1h at 4°C. The total protein concentrations of the membrane and soluble fractions were quantified using BCA Assay (Pierce, Thermo Scientific Ltd.) and stored in aliquots at -80°C until use [36].

### Two-dimensional Electrophoresis (2DE)

Immobilized pH gradient (IPG) strips, (11 cm, pH 5–8; BioRad, Hercules CA) were rehydrated for 16 h at 20°C in 200 μL of rehydration/sample buffer containing *B*. *burgdorferi* cytosolic extracts (approximately 250 μg of total protein). Isoelectric focusing (IEF) was carried out using the PROTEAN IEF (BioRad, Hercules CA) under the following conditions: Step 1, 250V for 20 min; Step 2, ramped to 8000 V over 2.5 h; and Step 3, 8000 V for a total of 20,000 V/h. Strips were then placed into equilibration buffer (EB) and disulfide groups were subsequently blocked with iodacetamide. Equilibrated IPG strips were then placed and fixed using hot agarose on the top of SDS-PAGE, 12% PAGE (Criterion Precast Gels; BioRad, Hercules CA) and separation of proteins in the second dimension done under reducing conditions. After electrophoresis, protein spots were visualized by staining with SYPRO Ruby gel stain (BioRad, Hercules CA) following manufacturer’s instructions. To assure maximal coverage, initial experiments were also performed with pH 3–10 IPG strips in the first dimension and SDS-12% PAGE gels in the second dimension [36]. These experiments revealed that most of the proteins of interest separated optimally between the pI of 5 and 8.

### Immunoblot analysis of oxidized proteins of *B*. *burgdorferi*

After loading the samples onto IPG strips and separating the proteins in the first dimension, oxidized proteins were labeled using the on-strip derivatization method [40]. Briefly, strips were incubated for 20 minutes in 10mM dinitrophenylhydrazine (DNPH) (Sigma-Aldrich) in 2M HCl at room temperature. After derivatization, strips were neutralized by incubating 30 minutes in EB-I followed by 30 minutes incubation in EB-II/iodoacetamide. Proteins were separated in the second dimension as described above. An OxyBlot protein standard (Chemicon International, Inc) was used in these gels as an internal control for the detection of oxidized proteins using the procedures described in this section. This protein standard was used as per manufacturer’s recommendations, and mixed with the same pre-stained molecular weight marker used in the SYPRO stained gels (EZ-Run Pre-stained *Rec* Protein ladder, Thermo Fisher Scientific Inc.). Separated proteins were transferred to PVDF membranes (Hybond GE Healthcare) using a semidry transfer unit (BioRad) for 30 minutes at 15V. Membranes were blocked overnight using 10% skim milk in Tris Buffer Saline (50mM Tris, 150mM NaCl) containing 0.2% Tween 20 (TTBS). After blocking, the membranes were incubated with 1:4000 dilution of the rabbit anti-DNP antiserum (Sigma-Aldrich) at room temperature for 2 hrs. This antiserum detects proteins that are preferentially derivatized using dinitrophenylhydrazine (DNPH). Membranes were washed 4 times with TTBS and 1:20,000 dilution of the secondary HRP-conjugated goat anti-rabbit antiserum (GE Healthcare) was added and incubated for 1hr at room temperature. Finally, membranes were washed 10 times in TTBS and oxidized proteins were visualized using Enhanced Chemiluminescence Plus Detection reagents (GE Healthcare). Spots corresponding to proteins with significant differences in the levels of oxidation in parental and *sodA* mutant strains were excised from SYPRO Ruby stained gels and subjected to mass spectrophotometry to determine the identity of the proteins oxidized in the presence of MV. The protein spots subjected to mass spectrometry analysis were those consistently detected in each biological replicate performed in this study.

### Identification of oxidized proteins by mass spectrophotometry

Spots of interest were excised from the gels and digested *in situ* with trypsin (Promega) in 40mM NH_4_HCO_3_ at 30°C overnight according to standard protocols [41–44]. The resulting digests were analyzed by capillary HPLC-electrospray ionization tandem mass spectrometry (HPLC-ESI/MS/MS) on a Thermo Fisher LTQ ion trap mass spectrometer fitted with a New Objective PicoView nanospray source. On-line HPLC separation of the proteolytic peptides was accomplished with an Eksigent Nano-LC micro HPLC system: column, PicoFrit (New Objective; 75 μm i.d.) packed to 10 cm with C18 adsorbent (Vydac; 218MSB5; 5 μm, 300 Å); mobile phase A, 0.5% acetic acid/0.005% TFA; mobile phase B, 90% acetonitrile/0.5% acetic acid/0.005% trifluoroacetic acid; linear gradient of 2 to 42% B in 30 min; flow rate, 0.4 μl/min. As a part of the data-dependent acquisition protocol, the seven most intense ions in each survey scan was sequentially fragmented in the ion trap by collision-induced dissociation using an isolation width of 2.5 and a relative collision energy of 35%. For identification of sites of protein modification, experiments were also conducted in which specific ions are targeted for MS^n^ analysis. Uninterpreted tandem mass spectra were analyzed by Mascot (Matrix Science, London, UK; 10 processor; in-house license). Methionine oxidation was selected as a variable modification for all searches. In some cases, oxidation of Cys (sulfinic, sulfenic and sulfonic acids), His and Trp was also considered. In addition, use of the "error-tolerant" search feature of Mascot was used to detect unanticipated modifications. Cross correlation of the Mascot results with X! Tandem, and determination of probabilities of protein identifications was determined using Scaffold (Proteome Software, Portland, OR). Assignment of the MS/MS fragments was verified by comparison with the predicted ions generated *in silico* by GPMAW (Lighthouse Data, Odense, Denmark).

### Purification of recombinant glyceraldehyde 3-phosphate dehydrogenase (rGAPDH) and generation of anti-GAPDH serum

Recombinant GAPDH (BB0057) with a C-terminal 6X histidine tag was over-expressed by inducing *E*. *coli* expression host Rosetta-gami containing pET23a/*bb0057* with 1mM IPTG for 3 hrs [19]. The purification of rGAPDH was done as described previously [19] and the purified rGAPDH was used to generate anti-serum in BALB/c mice in accordance with protocols approved by the Institutional Animal Care and Use Committee at UTSA.

### Immunoblot analysis

Total protein lysates from the parental, *sodA* mutant and complemented strains were separated using 12% SDS-PAGE gels, transferred to PVDF (Hybond-P GE Healthcare) membranes and blocked overnight using 10% skim milk in TTBS. The membranes were incubated for 1h at room temperature with anti-sera against OspA, OspB, OspC, DbpA, BBK32, BosR, lactate dehydrogenase (LDH), FlaB (as a loading control) and RpoS at appropriate dilutions [45–47]. The blots were developed following incubation with appropriate dilutions of HRP conjugated anti-mouse or anti-rabbit secondary antibodies using ECL Western blotting reagents (GE Healthcare, Buckinghamshire, UK). The same protocol was used for the detection of GAPDH (glyceraldehyde 6-phosphate dehydrogenase) before and after parental and the *sodA* negative strains were treated with MV.

### ATP levels

Spirochetes were grown to a cell density of 5x10^7^ cells/ml washed and treated with 20mM MV as described above. The lysates were prepared in buffer without DTT. Cell lysates were cleared by centrifugation at 10,000g for 15 minutes and supernatants were stored as aliquots at -80°C until use. Protein concentration of each supernatant was quantified using BCA assay (Pierce, Thermo Scientific, Ltd.). Levels of ATP in the parental and *sodA* mutant before and after oxidative stress were measured using the Adenosine 5'-triphosphate (ATP) Bioluminescent Assay Kit (Sigma-Aldrich). Briefly, a standard solution of ATP was prepared by making serial dilutions ranging from 10^-3^M to 10^-11^M of ATP to quantify the test samples. The assay was performed in a white, flat bottom, 96-well plate where 25μl of the ATP assay mix (provided by the manufacturer) were added per well and incubated at room temperature for 3 minutes. This was followed by the addition of 25μl of each of the standards and samples in triplicate to the wells containing the ATP assay mix rapidly and luminescence was measured using Synergy HT (BioTek) plate reader. ATP concentrations were calculated from a standard curve as picomoles (pM) and normalized per μg of protein. Levels of ATP were measured in four independent experiments. Values are represented as mean ± SD.

### NAD^+^-NADH levels

*B*. *burgdorferi* wild type and *sodA* mutant strains were grown to a cell density of 5x10^7^ cells/ml washed and treated with 20mM MV as described above. Levels of NAD^+^ and NADH were calculated using the Fluoro NAD/NADH kit (Cell Technology) following manufacturer’s recommendations. Briefly, after washing cells with HBSS thrice to remove MV and excess BSA, each sample was split into two aliquots one for NAD^+^ and the other one for NADH extraction. Washed cells were re-suspended in 200μl of the NAD^+^ or NADH extraction buffer and 200μl of the NAD/NADH lyses buffer was added to each tube. Samples were homogenized and incubated at 65°C for 15 minutes, cooled on ice and 100μl of reaction buffer was added. Each sample was then neutralized by the addition of 200μl of the opposite extraction buffer as per manufacturer’s instructions. Extracts were cleared by centrifuging at 8,000*g* for 5 minutes and 4°C and supernatants were used immediately in the fluorescence assay. An NADH standard solution was prepared by serial dilution ranging from 1000nM to 15.625nM. The assay was performed in a black, clear bottom, 96- well plate. 50μl of each sample and standard were added in triplicates followed by the addition of 100μl of reaction cocktail provided by the manufacturer. Plates were incubated at room temperature in the dark for 1hr and read with excitation at 560nm and emission at 590nm. NAD^+^ and NADH concentration were calculated from the calibration curves and normalized per μg of protein. NAD/NADH levels were obtained from 4 independent experiments. Values are represented as mean ± SD.

### Infectivity analysis of *sodA* mutant in C57BL/6, gp91/*phox* ^*−⁄−*^ and iNOS^*−⁄−*^ mice

Groups of mice (*n* = 3) were challenged intradermally with the 10^3^ or 10^5^ parental (ML23/E22) or *sodA* ^−^ mutant (*sodA* ^–^/E22) strain and the levels of infection were monitored after 21 days post-infection by either propagating different tissues (skin, lymph nodes, spleen, bladder, heart and one tibio tarsal joint) in BSKII growth medium or by enumeration of spirochetal numbers from a portion of skin, lymph node, spleen and one tibio-tarsal joint by quantitative real time PCR. Tissue samples propagated in borrelial growth medium were blind-passed into fresh medium after 5 days and spirochetal cultures were examined under dark field microscopy for presence of viable spirochetes. The levels of spirochetal DNA in each sample was normalized against mouse actin and represented as number of borrelial *flaB* copies for every 100, 000 actin copies.

### Statistical analysis

Levels of ATP, NAD^+^ and NADH in the wild type and *sodA* mutant before and after oxidative stress were compared by using one-way ANOVA, followed by the Bonferroni’s multiple comparison test.

## Results

### Increased oxidation of soluble proteins in the *sodA* mutant

**Fig 1:**
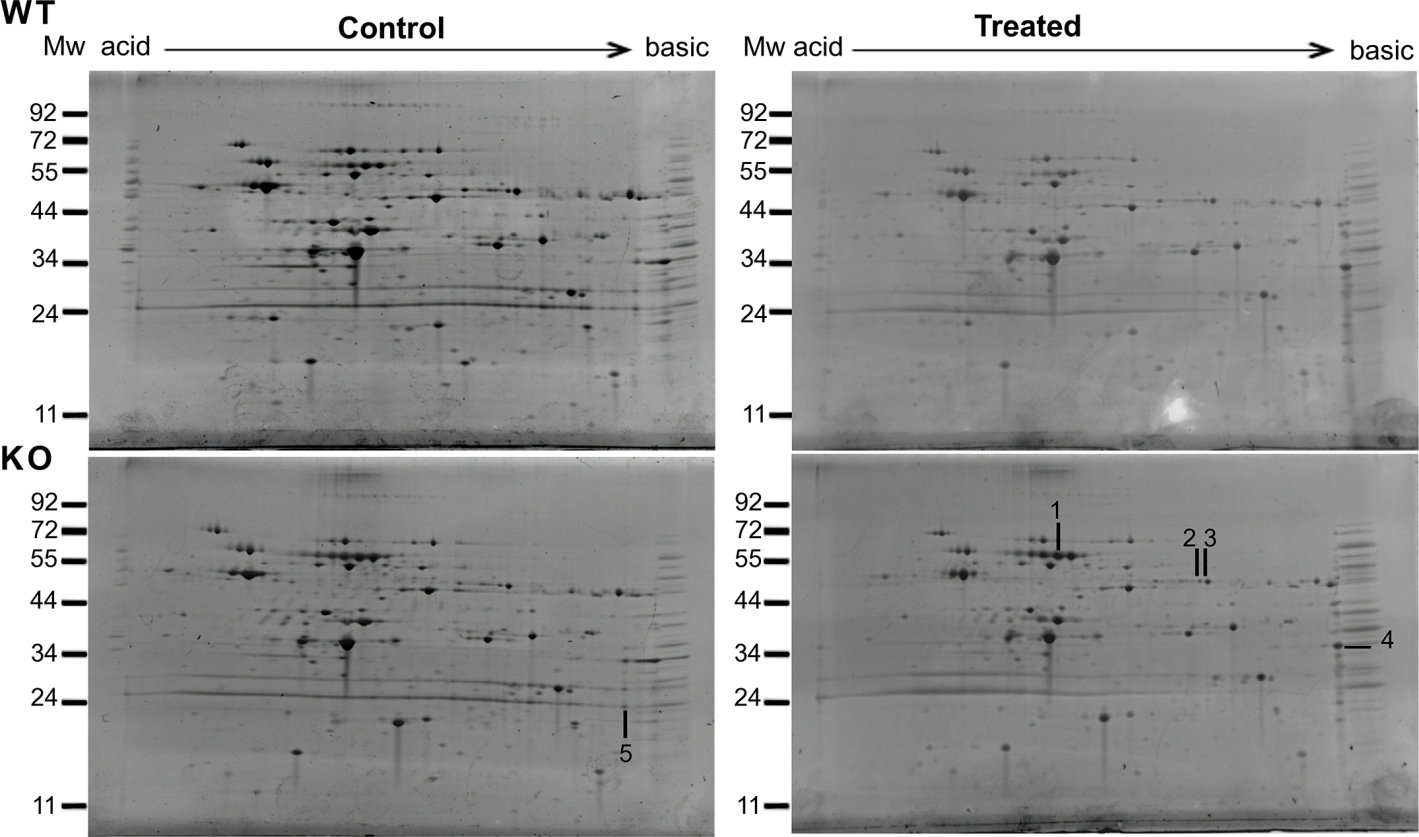
Two- dimensional electrophoretic (2-DE) profile of the soluble fractions of parental and *sodA* mutants strains of *B*. *burgdorferi*. Soluble proteins of *B*. *burgdorferi* parental (wt) and *sodA* mutant (mt) strains before (Control) and after exposure to 20 mM MV (Treated) were separated from membrane proteins by ultracentrifugation and resolved using IPG strips (pH 5–8) in the first dimension and by SDS-12% PAGE gels in the second dimension. Following 2-DE, the total proteins were stained with SYPRO Ruby (Molecular Probes) with basic and acid ends indicated for each gel. Numbers with lines represent the spots that correlate with oxidized proteins detected by immunoblot in Fig 2. Spots were excised and analyzed by MALDI-TOF MS. Results are outlined in Table 1 with spot number indicated within parenthesis. Molecular mass markers (MW) are represented on the left side of each gel in kDa. Representative gels are presented in this figure.

**Fig 2:**
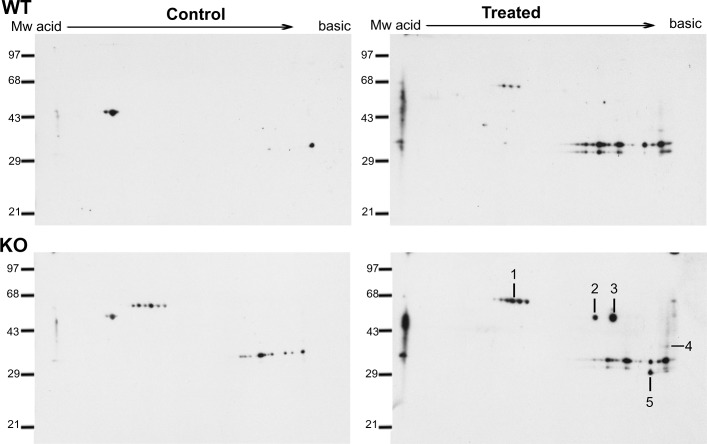
Immunoblot analysis of oxidized soluble proteins of parental and *sodA* mutant strains of *B*. *burgdorferi*. Soluble proteins from the parental strain (wt) and *sodA* mutant (mt) were obtained before (Control) and after treatment with 20 mM MV (Treated) and subjected to two-dimensional gel electrophoresis after derivatization with dinitrophenylhydrazine (DNPH) as described in the material and methods. The proteins were transferred to PVDF membranes and probed with anti-DNP antiserum and blots developed using Enhanced Chemiluminescence Plus Detection reagents. Lines and numbers represent the protein spots oxidized in the *sodA* mutant compared with the parental strain. Spots were excised from the SYPRO stained gels as shown in Fig 1 and analyzed by MALDI-TOF MS. Molecular mass markers (MW) are represented on the left side of each gel in kilodaltons, OxyBlot Protein standard was used in this assay (Chemicon International, Inc.). Representative blots are presented in this figure.

**Fig 3:**
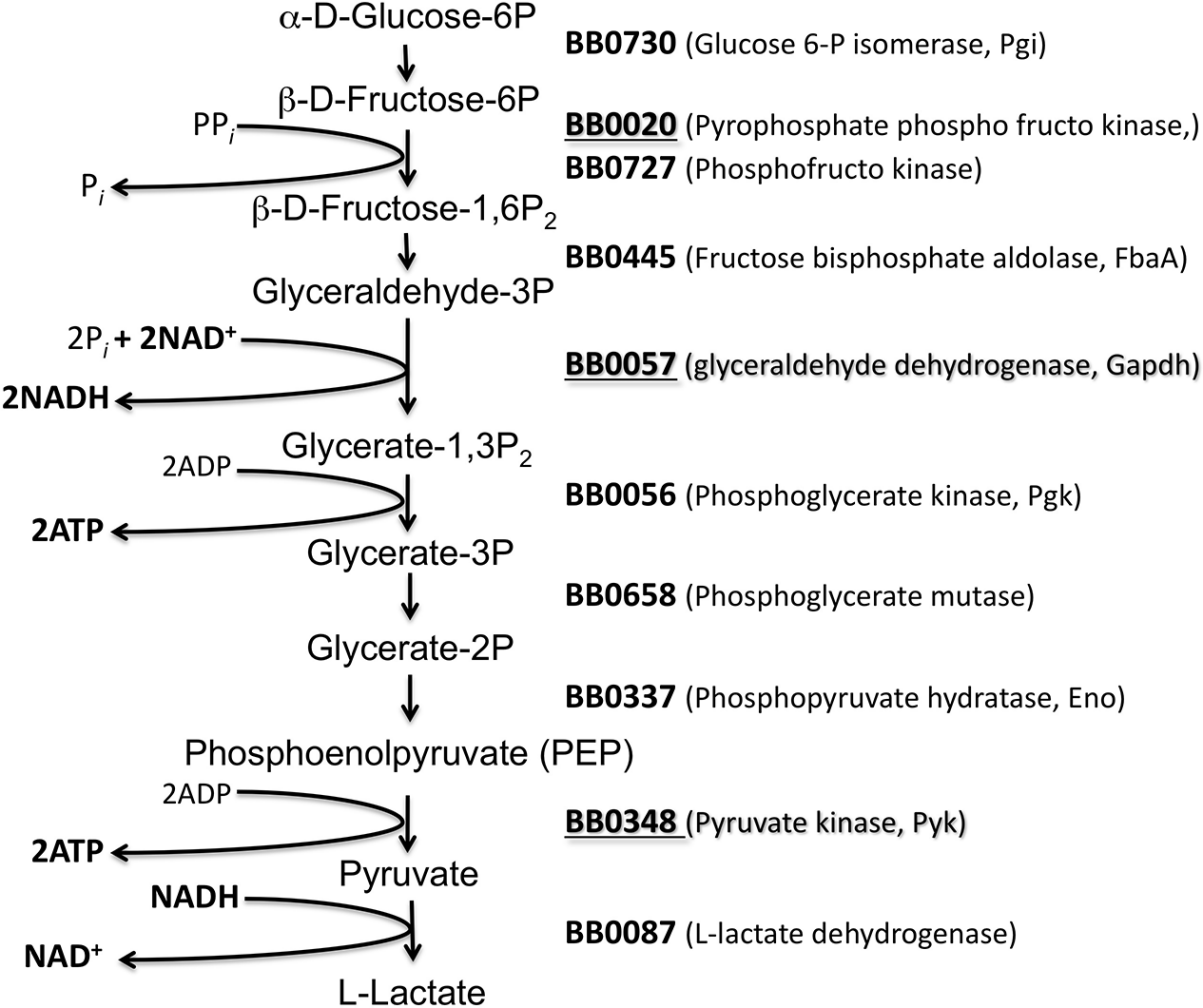
Glycolytic pathway of *B*. *burgdorferi*. A schematic representation of the glycolytic pathway based on the genome information from Frazer et al (1997). Numbers indicate the locus in *B*. *burgdorferi* encoding the enzyme catalyzing each of the steps of the pathway. Highlighted in bold and underlined are the enzymes with oxidative damage in the *B*. *burgdorferi sodA* mutant strain after treatment with the MV.

We hypothesized that lack of *sodA* will impact the functions of cytosolic proteins critical for multiple metabolic pathways of *B*. *burgdorferi*. Therefore, we exposed the parental and mutant strains to methyl viologen and determined the identity of soluble borrelial proteins that were oxidized using anti-DNP antibodies following derivatization of oxidized proteins using dinitrophenylhydrazine (DNPH) [36]. As shown in Fig 1, there were no significant differences in the levels of proteins stained with SYPRO Ruby between the parental and mutant strains before and after treatment with methyl viologen. On the other hand, as shown in Fig 2, immunoblot analysis using anti-DNP antibodies showed a collection of proteins with significant levels of oxidation in the *sodA* deficient strain compared to the parental control strain. Furthermore, even in the absence of MV treatment, the *sodA* mutant exhibited increased levels of oxidation in select proteins that were not observed in the parental strain. Five proteins that were preferentially oxidized in the *sodA* mutant were identified by mass spectrometry (Table 1). Three of the five identified proteins [glyceraldehyde 6-phosphate dehydrogenase (BB0057, GAPDH), pyrophophate phospho fructo kinase (BB0020, Pfk) and pyruvate kinase (BB0348, Pky)] are part of the glycolytic pathway (Fig 3). One heat shock protein (hsp90, HtpG, BB0590) involved in protein folding and the outer surface protein A (OspA, BBA15) known to play a key role in the attachment of the spirochetes to the tick midgut were also observed to have increased levels of oxidation in the *sodA* negative mutant [48]. We also analyzed the putative target residues of the enzymes that were identified by mass spectrophotometric analysis and determined that the active sites of several of these enzymes had residues that could serve as targets of oxidation (Table 2). These observations demonstrated that oxidation of key residues of cytosolic proteins are increased in the absence of *sodA* and that these changes could lead to alterations in the survival capabilities of *B*. *burgdorferi* in the presence of oxidative stressors. We focused on identifying the effects of oxidation of soluble, cytosolic proteins on the central metabolic pathways of *B*. *burgdorferi* although determination of levels of oxidation of membrane proteins may provide additional information on the phenotype of the *sodA* mutant. In addition, the availability of specific antibody reagents to major lipoproteins of *B*. *burgdorferi* also allowed us to determine if there are significant changes in the levels of expression of select borrelial proteins as described below.

**Table 1:** Proteins with increased levels of oxidation in the *sodA* mutant identified by mass spectrometric analysis.

			Peptides identified		Coverage (%)			
Protein ID*	pI	Size (KDa)	Exp 1	Exp 2	Exp 1	Exp 2	Gene Locus	Function
• Glyceraldehyde 6-phosphate dehydrogenase (GAPDH) (4)	8.2207	36.25	4	15	34	45	*bb0057*	Glycolysis
• Pyrophophate phospho fructo kinase (2)	6.5319	62.48	9	11	14	20	*bb0020*	Glycolysis
• Pyruvate kinase (Pky) (3)	7.23	53.03	22	5	32	10	*bb0348*	Glycolysis
• Chaperon hp90 (HtpG) (1)	5.3925	75.43	14	15	22	25	*bb0560*	Protein folding
• Outer Membrane protein A(5)	9.35	29.35	7	ND	27	ND	*Bba015*	Cell envelop, Lipoprotein

*: The numbers of the spots in the gel shown in Fig 2 are indicated within parentheses.

ND: Not determined

**Table 2:** Amino acid residues that are potential targets for carbonylation within the oxidized proteins.

Protein ID	Position^1^	Sequence^2^	Function
Glyceraldehyde 6-phosphate dehydrogenase (GAPDH, *bb0057*)	7–12	GFGR**I**G	ATP binding site
	148–164	SNA S**C**TTN**C** **L**A**PL** A**K**V**L**	Active site with C-xxx-C motif
	179–203	**H**A YTNDQ**RIL**D**L PH**SD**LRR**A**R**A AA**L**	S-Loop
Pyrophophate phospho fructo kinase (*bb0020*)	280–283	**KKK**T	AMP phosphorylation site
Pyruvate kinase (Pky, *bb0348*)	209–221	**VK II**S**KI**ENQEG **I**	Phosphokinase activity
Chaperon hsp90 (HtpG, *bb0560*)	21–30	YS**HK**E**I**F**LR**E	Signature site
	3–238	Multiple target residues could be potentially oxidized	ATPase activity site for Hsp90 proteins

1: amino acid position from N-terminal end of the protein.

2: The possible target residues for carbonylation are indicated in the bold: cysteine (C), histidine (H), leucine (L), isoleucine (I), valine (V), proline (P), arginine (R) and lysine (K).

### Levels of ATP are reduced in the *sodA* mutant

**Fig 4:**
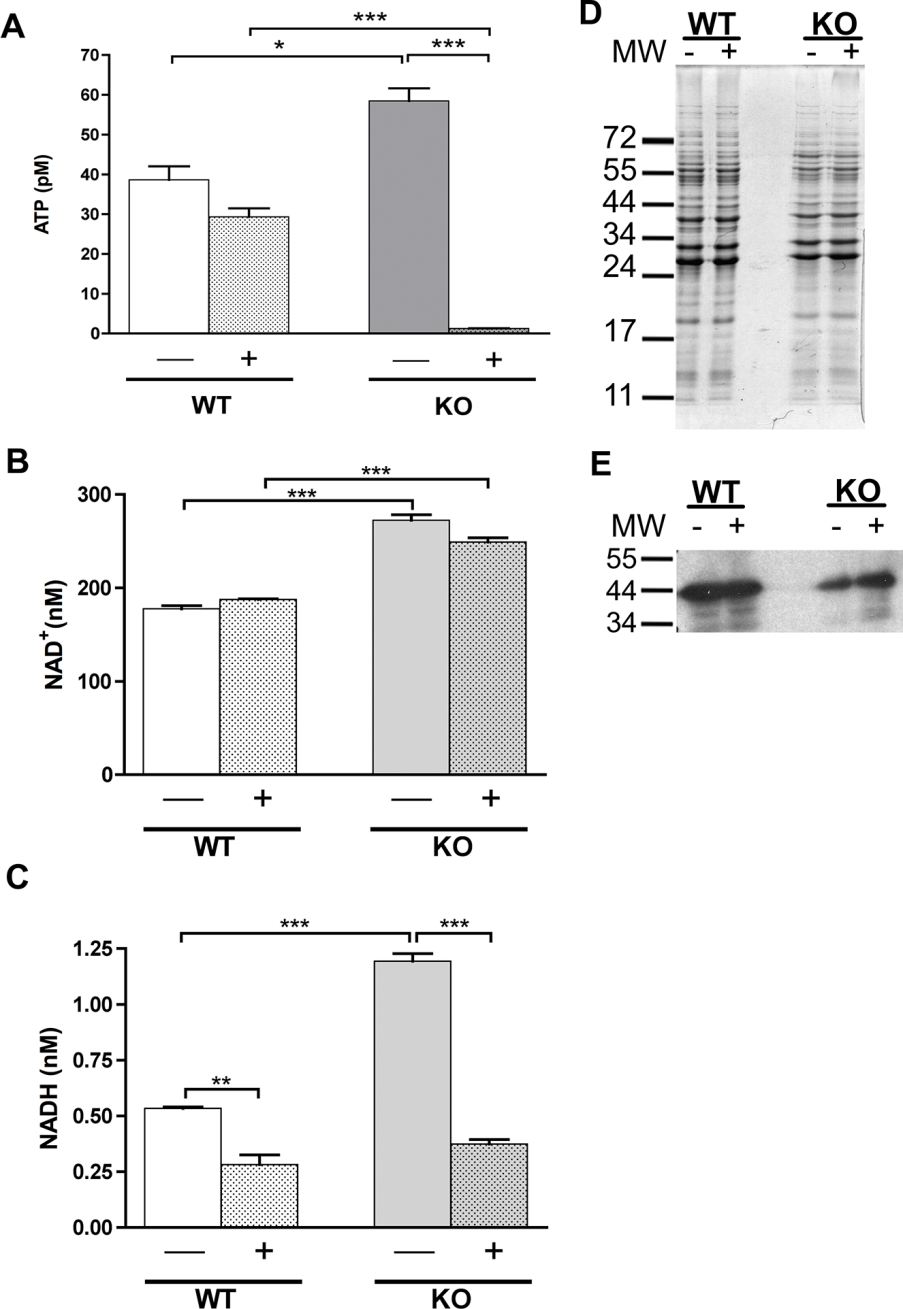
Levels of ATP, NAD/NADH and GAPDH in parental and *sodA* mutant strains of *B*. *burgdorferi*. Soluble proteins from the parental (wt) and *sodA* mutant (mt) of *B*. *burgdorferi* before (-) and after (+) treatment with 20 mM MV were prepared for measuring the ATP levels using ATP-Bioluminescent Assay Kit as per manufacturer’s instructions and the luminescence was measured using Synergy HT (BioTek) **(A)**. A similar procedure was adopted for the isolation of soluble proteins for measurement of NAD/NADH using Fluoro NAD/NADH kit (Cell Technology) **(B and C)**. The data represented were from 4 separate experiments and statistical analysis was carried out using one-way ANOVA followed by the Bonferroni’s multiple comparison test. * Denotes *P* value < 0.05, ** *P* value < 0.01 and *** *P* value < 0.001. The soluble proteins were from the parental (wy) and the *sodA* mutant (mt) were isolated as described above before (-) and after (+) treatment with 20 mM MV and separated on SDS-12% PAGE gels and stained with Coommassie blue **(D)** or transferred to PVDF membrane, probed with anti-GAPDH serum and blots developed using Enhanced Chemiluminescence system **(E).** Molecular masses in kilodaltons are indicated to the left of panel D and E.

GAPDH, Pfk and Pky are key enzymes of the glycolytic pathway (Fig 3). Oxidation of these proteins might alter their function and consequently, there is a possibility that the cellular levels of ATP could be reduced rendering the *sodA* mutant more susceptible under conditions of limited nutrient availability or in microenvironments with limited energy sources [49–54]. To test this hypothesis, we measured the levels of ATP before and after treatment with 20mM MV in the parental and *sodA* negative strains. As shown in Fig 4A, in the presence of MV, the levels of ATP in the *sodA* negative strain were significantly reduced when compared with those of the untreated sample (*P* < 0.001), and were also lower than the levels observed in the parental strain after treatment (*P* < 0.001). On the other hand, the ATP levels in the parental strain were not significantly reduced in the presence of stressor. Furthermore, we hypothesized that if levels of ATP are reduced, levels of NADH will also be affected by the increased oxidation of GAPDH, Pfk and Pky in the *sodA* mutant with an increase in the levels of accumulated NAD^+^ (Fig 3). As shown in Fig 4B, the parental strain had the same levels of NAD^+^ in the presence or absence of MV, while the *sodA* mutant strain had significantly more NAD^+^ compared to the parental strain independent of the levels of oxidative stress. This accumulation of NAD^+^ is consistent with the observation that GAPDH—a critical enzyme involved in generating NADH from NAD^+^—presumably has reduced enzymatic functions due to oxidation of critical residues in its active site. We observed a reduction in the levels of NADH in the parental strain after treatment with MV (*P* < 0.01) as a potential outcome of oxidation of GAPDH in wild type strain (Fig 4C). Surprisingly, the levels of NADH in the *sodA* negative strain were significantly higher than those observed in the parental strain (*P* < 0.001) prior to treatment with MV. However, the levels of NADH in the *sodA* mutant after treatment with MV dropped to levels significantly lower than those observed in the untreated sample (P < 0.001) as well as that observed in the treated parental strain. These observations suggest that oxidative damage of key enzymes, notably GAPDH, involved in the glycolytic pathway, result in a significant reduction of the energy levels of the spirochetes and could translate into reduced survival capability of the *sodA* mutant in select microenvironments. The survival capabilities of *sodA* mutant of *B*. *burgdorferi* could be significantly compromised under conditions of increased oxidative stress due to a reduced energy flux and contribute to a defect in the colonization of the mouse model of Lyme disease [19].

### Levels of select lipoproteins in the *sodA* mutant

**Fig 5:**
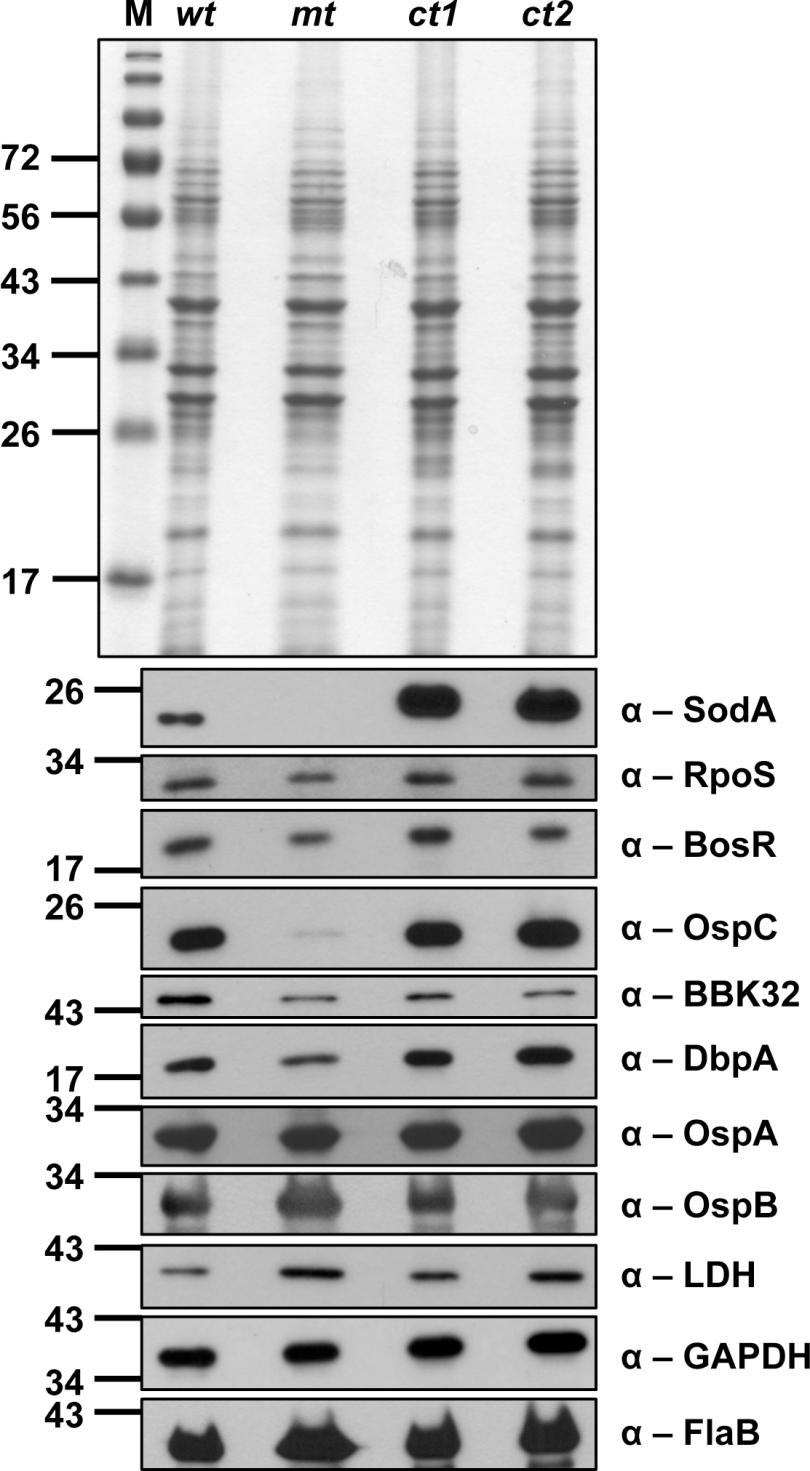
Levels of virulence determinants in *B*. *burgdorferi* lacking SodA. The parental (wt), *sodA* mutant (mt), the two complemented strains (ct1 and ct2) were propagated at 32°C at pH7.6 and total protein lysates were separated on SDS-12%PAGE either stained with Coomassie blue **(A)** or transferred to PVDF membranes for immunoblot analysis using antisera against different borrelial proteins indicated to the right of each blot **(B).** Note the reduction in expression of RpoS, decorin binding protein A (DbpA), fibronectin binding protein (BBK32) and outer surface protein C (OspC) in the *sodA* mutant compared with the control strains. No obvious differences were noted in the levels of OspA, OspB. Lactate dehydrogenase (Ldh) and FlaB levels between the *sodA* mutant and control strains. Molecular masses (MW) in kilodaltons are indicated to the left of each blot.

In order to determine the changes in the levels of lipoproteins in the *sodA* mutant, total protein lysates from parental (ML23/E22), *sodA* mutant and two complemented strains propagated under conventional growth conditions (32˚C/ pH7.6) were separated on SDS-12%PAGE gels. Total staining of the proteins with Coomassie brilliant blue showed equivalent loading of proteins (Fig 5A) and immunoblot analysis was done following electrotransfer of separated proteins using specific sera against select lipoproteins of *B*. *burgdorferi* (Fig 5B). The levels of DbpA, BBK32 and OspC were reduced in the *sodA* mutant compared to the control strains. However, the levels of OspA, OspB (lipoproteins) and FlaB (major flagellin, loading control) and GAPDH were similar in all three strains (Fig 5B). Lactate dehydrogenase, however was relatively more in the *sodA* mutant compared to the wild-type strain. The levels of RpoS and BosR was lower in the *sodA* mutant compared to the control strains suggesting that the reduction in the levels of select lipoproteins may be reflective of the reduction in the levels of RpoS as these lipoproteins are part of the *rpoS* regulon [12, 55–57]. While DbpA and BBK32 bind to mammalian extracellular matrix components such as decorin and fibronectin respectively OspC is required for infectivity of mammalian host [58–62]. OspA/B is up-regulated under tick-specific conditions whereas OspC, DbpA and BBK32 are up-regulated under mammalian host-specific conditions and therefore a reduction in the levels of these key lipoproteins likely contributed to the reduced levels of infectivity of the *sodA* mutant [60, 61].

### *sodA* mutant is incapable of survival in gp91/*phox*- and iNOS-deficient strains of mice

**Fig 6:**
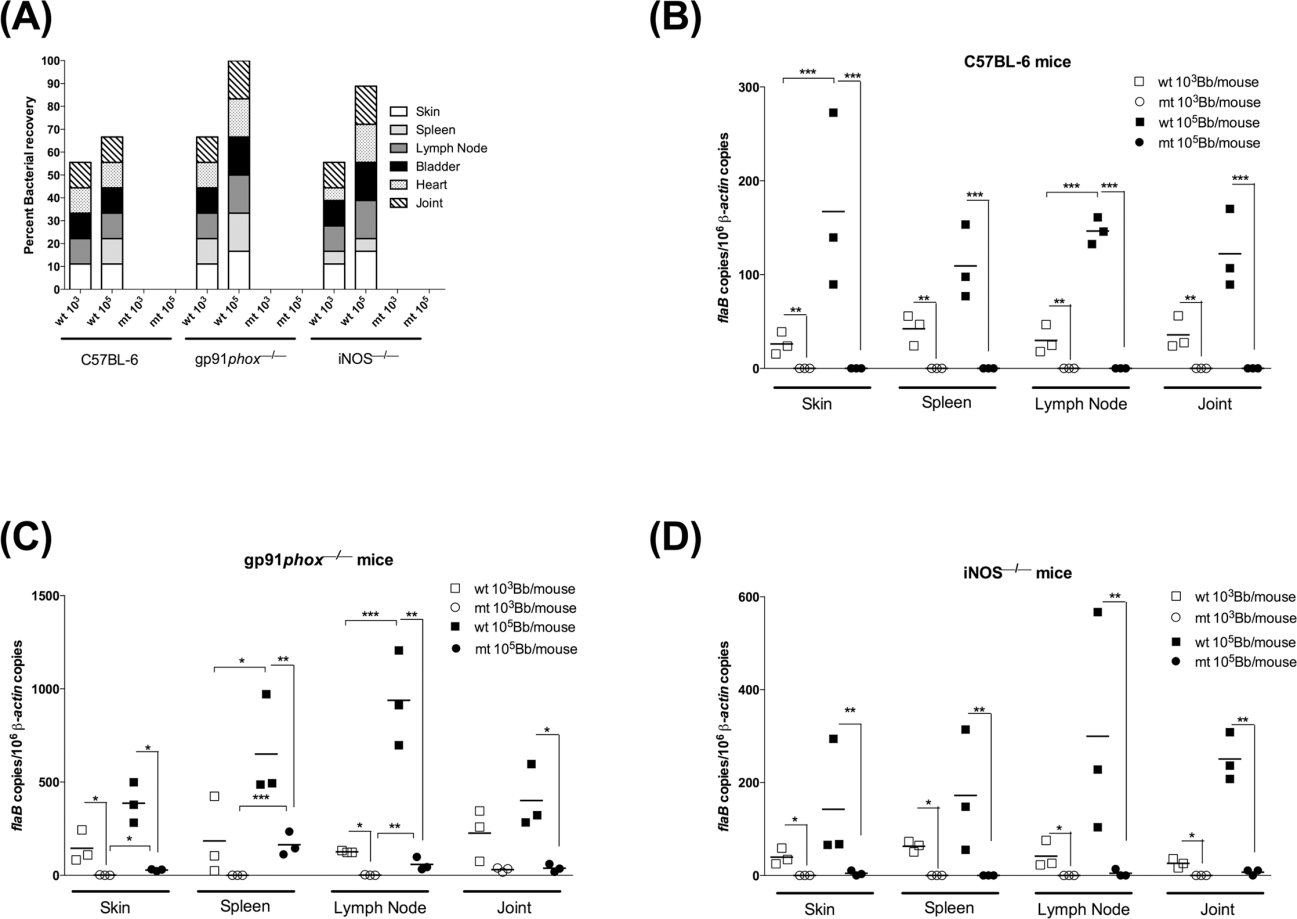
*sodA* mutant is unable colonize gp91/*phox* ^*–/–*^ and iNOS^–/–^deficient mice. Groups (*n* = 3) of mice were infected with either parental (wt) or *sodA* mutant (mt) at 10^3^ or 10^5^ spirochetes per mouse. **(A)** Twenty one days post-infection, samples of skin, spleen, one popliteals lymph node, bladder, heart and one tibio-tarsal joint were aseptically isolated and propagated in BSK-II medium with supplemented with 6% normal rabbit serum and scored for viable spirochetes using dark field microscopy. The y axis indicates the total number of tissues tested that are positive for infection with each rectangular box representing a specific tissue and the height of each box is indicative of the number of animals with infection in the respective tissue. **(B-D)** Quantitative real-time PCR analysis of spirochetal burden in infected mouse tissues. Skin, spleen, lymph nodes and joint were isolated at 21 days post-infection from groups of C57BL/6, gp91/*phox* ^*−⁄−*^ or iNOs^−⁄−^ mice. The number of spirochetes in these tissues at the end of infection was enumerated by quantitative real-time PCR. Numbers of borrelial *flaB* copies were normalized to mouse β-actin copies and levels of significance were as follows: **, *P*<0.01; *, P<0.05.

We have previously shown that even though borrelial DNA from *sodA* mutant was detected in different tissues following intradermal challenge of C3H/HeN mice, we were unable to isolate viable spirochetes from any of the tissues tested at 21 days post-infection [19]. We hypothesized that the inability of the *sodA* mutant to survive in C3H/HeN mice could be due to one or more oxidative stressors such as reactive oxygen specifies (ROS) or reactive nitrogen species (RNS). Therefore, we evaluated the phenotype of *sodA* mutant in either gp91/*phox* or iNOS- deficient mice lacking ROS and RNS production, respectively, following intradermal needle challenge at either 10^3^ or 10^5^ organisms per mouse. We were unable to re-isolate viable *sodA* mutant spirochetes in all the tissues tested even though *sodA* mutant-specific DNA was detected (Fig 6). Viable wild-type spirochetes (ML23-E22) were isolated from all tissues from 2 out of 3 C57BL/6 (wild type mouse strain), while they were isolated from all tissues from gp91/*phox* and iNOS-deficient mice (Fig 6A). In addition, qPCR data (Fig 6B, 6C and 6D) shows that the bacterial burden of gp91/*phox* ^*—/—*^ and iNOS^*—/—*^ mice infected with the parental strain was higher than the bacterial burden of C57BL6 mice infected with the same strain. In addition, there was increased survival of the parental strain in mice deficient in generating reactive oxygen species (ROS, gp91/*phox* ^*—/—*^ mice, Fig 6C), compared to reactive nitrogen species (iNOS^*—/—*^ mice, Fig 6D). Based on these observations, it appears that the *sodA*-deficient spirochetes are incapable of survival in the presence of either reactive oxygen or reactive nitrogen species even though we were able to detect low levels of borrelial DNA in select tissue samples from transgenic mice deficient in ROS or RNS.

## Discussion

The ability of *B*. *burgdorferi* to survive in the mammalian host requires not only a coordinated regulation of expression of a variety of determinants to overcome the deleterious effects of the adaptive immune system, but also mechanisms to survive and colonize in microenvironments that are limited in nutrients and elevated in mediators of innate immune response [63]. Some of the components of the innate immune systems such as complement, phagocyctic and bacteriocidal effects of activated macrophages and neutrophils, as well as the appropriate activation of the Toll-like receptors, regulate the host-pathogen interactions either facilitating clearance of the bacteria or allow for successful colonization leading to establishment of the disease [1]. These interactions are critical for *B*. *burgdorferi*, which is dependent on the host to acquire a variety of nutrients due to its limited metabolic capabilities. Therefore the limited metabolic capabilities of *B*. *burgdorferi* and/or accumulation of lesions in the DNA, proteins and lipids can significantly reduce the *in vivo* survival capabilities of Lyme spirochetes [64].

The lack of requirement of iron as a cofactor for enzymes and the presence of very limited concentrations of free Fe in *B*. *burgdorferi* has been shown to limit the deleterious effects of the Fenton reaction on DNA. On the other hand, much of the damage due to oxidative stressors were directed at polyunstaturated fatty acids acquired from the host or *in vitro* growth medium [20, 21, 26, 27, 65]. Among the polyunstraturated fatty acids linoleic acid exhibited increased oxidation leading to detectable damage in the borrelial membranes [27]. In addition to induced damage to membranes through lipid peroxidation, MV a charged quaternary ammonium compound (MV^2+^) can diffuse through bacterial outer membrane when reduced to a more stable radical with a single positive charge (MV^+^) [66]. Moreover, previous studies have shown that MV can be accumulated in *Salmonella enterica* serovar Typhimurium, in which a number of porins have been characterized as efflux pumps that can move MV out of the cell and, therefore induce resistance to oxidative damage [67, 68]. In a previous study, we found that increased intracellular accumulation of O_2_^—^ following treatment with MV, resulted in increased sensitivity of the *B*. *burgdorferi sodA* deficient mutant, compared to the parental or complemented strains [19]. Furthermore, the regulation of gene expression under oxidative stress has been reported in several publications [20, 21, 65, 69–73]. In our study, proteomic analysis of the soluble fraction from the *sodA* mutant demonstrated a significant increase in the levels of oxidation as determined by immunoblot analysis using antibodies specific to DNP-derivatized proteins (Figs 1 and 2). Mass spectrophotometric analysis of the corresponding proteins from SYPRO Ruby stained gels revealed that several oxidized proteins (GAPDH, phospho-fructo-kinase and pyruvate kinase, Fig 3 and Table 1) were critical enzymes involved in glycolysis and if these proteins were to be oxidized, there could be a concomitant decrease in the levels of ATP generated. Consistent with this prediction, the levels of ATP and NADH were significantly reduced in the *sodA* mutant treated with MV compared to the untreated sample (*P*<0.001). Moreover, there was also significant decrease in the levels of ATP in the *sodA* mutant compared to the parental control strain treated with MV (*P*<0.001) even though the untreated *sodA* mutant had higher levels of ATP (P<0.05) compared to its parental counterpart. While this difference in the initial levels of ATP could be reflective of multiple metabolic parameters such as growth, motility and other energy consuming functions that may deplete the levels of ATP in the parental strain, the most significant difference in ATP levels were observed following the treatment of the *sodA* mutant with MV (Fig 4A). Moreover, the levels of NAD^+^ was similar in both the parental and *sodA* mutant with and without treatment while there was a significant decrease in the levels of NADH following the treatment of *sodA* mutant with MV (Fig 4B and 4C). One of the steps of the glycolytic pathway that contributes to the generation of NADH + H^+^ from NAD^+^ + Pi is the oxidation and phosphorylation of glyceraldehyde- 3- phosphate (GAP) to 1, 3 bisphosphoglycerate by glyceraldehyde-3-phophate dehydrogenase (GAPDH). This suggests that the consequence of oxidation of GAPDH could result in reduced amount of NADH due to the alteration in the active sites of GAPDH following treatment of the *sodA* mutant with MV. Following the same line of evidence, previous studies with *Pseudomonas aeruginosa* demonstrated that, in the absence of functional intracellular GAPDH there was an increase in sensitivity to oxidative stress generated by MV [74].

Analysis of the amino acid sequence of GAPDH also revealed that there are several residues, most notably cysteines in the active site (CXXXC within the residues 148 to 164; Table 2), that could have a significant effect on the enzymatic function of GAPDH. It needs to emphasized that while the mass spectrophotometric analysis facilitated identification of the proteins with increased oxidation, the functional deficiency of GAPDH could be due to changes in the key residues of the active site, ATP binding site or in the S-loop [53, 54]. Even though there was increased oxidation of GAPDH in the *sodA* mutant following treatment with MV, there was no significant difference in the levels of this proteins as determined by immunoblot analysis using monospecific antibodies (Fig 4E), suggesting that oxidative changes *per se* may not be sufficient for increased turnover of this protein. Similar modifications in the active sites of other oxidized proteins such as the AMP phosporylation site of phosphofructo kinase or the site involved in the phosphokinase activity associated with pyruvate kinase either individually or together could lead to significant reduction in the ATP generating capabilities of the spirochetes via the glycolytic pathway. Therefore, it is interesting to speculate that while the levels of ATP and NADH are higher in the untreated *sodA* mutant compared to the parental control strain, the induction of oxidative stress with MV leads to a significant reduction in the concentrations of both of these compounds due to the reduction in function of enzymes contributing to the energy generating phase (second phase) of glycolysis. It is important to state that the levels of ATP and NADH measured following addition of MV is essentially a snapshot of the energy flux of spirochetes and that it is feasible that under *in vivo* conditions a proportion of spirochetes may not have sufficient energy to mediate critical metabolic processes in select microenvironments. The accumulation of oxidized residues in the absence of a scavenger of superoxide generated within the cytoplasm could therefore lead to reduced or no survival capabilities for *B*. *burgdorferi* and this scenario could partly contribute to the lack of infectivity of the *sodA* mutant in the C57BL6 mice as well as in gp91/phox and iNOS-deficient mice respectively (Fig 6). Since RNS has synergistic effects with hydrogen peroxide to limit bacterial survival, we anticipated that the iNOS deficient mice could support the colonization of the *sodA* mutant due to the reduced levels of hydrogen peroxide formation consistent with the lack of dismutation reaction in the *sodA* mutant. It is feasible that the pH of select microenvironments could favor the formation of protonated OH^—^ions that could traverse through the membranes resulting in survival deficits [75, 76]. These observations expand the role of SodA in the patho-physiology of the Lyme spirochetes.

In addition to the effects of oxidation of key enzymes of the glycolytic pathway, there was increased oxidation of the HtpG or Hsp90 (BB0560) in the *sodA* mutant. This could lead to a variety of metabolic deficits such as improper of folding of proteins affecting their transport to appropriate locales of cell most notably to the periplasm. Since many of the lipoproteins critical for the pathogenic mechanisms of *B*. *burgdorferi* are located on the surface, the chaperone functions of HtpG may therefore be a key component of the adaptive response during the colonization of the mammalian host. We also observed that OspA was one of the proteins that exhibited increased oxidation in the *sodA* mutant suggesting that select residues of lipoproteins could be targets of oxidation during their synthesis in the cytosol. This could have implications in the level of surface expression of lipoproteins, which could limit the infectivity potential of the spirochetes.

Immunoblot analysis of lysates from spirochetes propagated at pH7.6/32°C revealed decreased synthesis of OspC, DbpA and BBK32 in the mutant compared to the parental and the complemented strains (Fig 5). Moreover, the levels of regulators of gene expression such as RpoS and BosR were also lower and partly explain the reduction in the levels of OspC, DbpA and BBK32—key virulence-associated lipoproteins that are members of its regulon [55, 57]. Recently, post-transcriptional and post-translational regulation of RpoS in *B*. *burgdorferi* is being investigated in greater detail. Dulebohn and others demonstrated that a plasmid-encoded protein, BBD18, contributes to post-transcriptional regulation of *rpoS* presumably via protein destabilization/degradation [77]. Even though *B*. *burgdorferi* has homologs of Clp protease complex (ClpXP), the absence of an apparent homolog of an adaptor protein, RssB, that has been shown to deliver RpoS to the Clp protease complex in other bacterial systems, suggests that either other borrelial proteins provide these adaptor functions or a novel mechanism contribute to regulating RpoS at post-transcriptional level [77–79] One other mechanism that leads to proteolysis of RpoS in *E*. *coli* is the levels of ATP (but not GTP or NADH) [80]. ClpXP is incapable of proteolysis of RpoS with low rates of ATP hydrolysis even though its proteolytic effects on other proteins remain intact [80]. Since the ATPs levels in the *sodA* mutant was significantly higher under normal laboratory growth conditions (pH 7.6 32°C), it is possible that the increased levels of ATPs could lead to increased proteolysis of RpoS resulting in reduced levels of virulence associated lipoproteins consistent with our data shown in Fig 5. While the levels of ATP are considerably reduced in the *sodA* mutant following treatment with MV, it can be argued that the lack of infectivity of the *sodA* mutant is a reflection of the cumulative effects of defects in a variety of proteins involved in both the pathogenic mechanisms and in the reduced efficiency of central metabolic pathways such as glycolysis resulting in reduced ATP/NADH flux. Moreover, the levels of BosR are also lower in *sodA* mutant compared to the control strains (Fig 5 α-BosR). BosR was previously shown to be a target for nitrosative damage induced in the presence of NO donor [28]. We are cognizant of the fact that the culture conditions of the strains grown under standard laboratory growth conditions (pH7.6/32°C), although consistent for all mutant and control strains, reflect the phenotype of the *sodA* mutant. Previous studies from our laboratory showed significant differences in the levels of regulators (RpoS, BosR) and a variety of lipoproteins (OspC, DbpA and BBK32) when *B*. *burgdorferi* cultures are shifted from growth conditions mimicking the mid-gut of the tick vector before (pH7.6/23°C) to after (pH6.8/37°C) the ingestion of a blood meal [45, 81–86]. Similar analysis of the functional status of proteins using multiple, rapidly expanding proteomic tools will facilitate identification of differences between the *in vitro* and *in vivo* phenotypes that are not readily discernable in pauci-bacillary infections such as that of *B*. *burgdorferi* in experiment models of infection[87–89]. In summary, the absence of *sodA* leads to not only changes in the functional roles of proteins critical for the central and intermediary metabolism but also play a role to connect the levels of small energy products such as ATP, NAD and NADH towards controlling levels of synthesis of key virulence determinants critical for infectivity of the vertebrate hosts.
